# Improved Canine and Human Visceral Leishmaniasis Immunodiagnosis Using Combinations of Synthetic Peptides in Enzyme-Linked Immunosorbent Assay

**DOI:** 10.1371/journal.pntd.0001622

**Published:** 2012-05-22

**Authors:** Míriam Maria Costa, Marcos Penido, Mariana Silva dos Santos, Daniel Doro, Eloísa de Freitas, Marilene Susan Marques Michalick, Gabriel Grimaldi, Ricardo Tostes Gazzinelli, Ana Paula Fernandes

**Affiliations:** 1 Departamento de Bioquímica e Imunologia, Universidade Federal de Minas Gerais, Instituto de Ciências Biológicas, Belo Horizonte, Minas Gerais, Brasil; 2 Departamento de Análises Clínicas e Toxicológicas, Universidade Federal de Minas Gerais, Faculdade de Farmácia, Belo Horizonte, Minas Gerais, Brasil; 3 Departamento de Parasitologia, Universidade Federal de Minas Gerais, Instituto de Ciências Biológicas, Belo Horizonte, Minas Gerais, Brasil; 4 Departamento de Imunologia, Fundação Oswaldo Cruz, Instituto Oswaldo Cruz, Rio de Janeiro, Rio de Janeiro, Brasil; 5 Centro de Pesquisas René Rachou, Fundação Oswaldo Cruz, Belo Horizonte, Minas Gerais, Brasil; 6 Division of Infectious Diseases and Immunology, University of Massachusetts Medical School, Worcester, Massachusetts, United States of America; Institut Pasteur de Tunis, Tunisia

## Abstract

**Background:**

Zoonotic visceral leishmaniasis (VL) is a severe infectious disease caused by protozoan parasites of the genus *Leishmania* and the domestic dogs are the main urban parasite reservoir hosts. In Brazil, indirect fluorescence antibody tests (IFAT) and indirect enzyme linked immunosorbent assay (ELISA) using promastigote extracts are widely used in epidemiological surveys. However, their sensitivity and specificity have often been compromised by the use of complex mixtures of antigens, which reduces their accuracy allowing the maintenance of infected animals that favors transmission to humans. In this context, the use of combinations of defined peptides appears favorable. Therefore, they were tested by combinations of five peptides derived from the previously described *Leishmania* diagnostic antigens A2, NH, LACK and K39.

**Methodology/Principal Findings:**

Combinations of peptides derived A2, NH, LACK and K39 antigens were used in ELISA with sera from 44 human patients and 106 dogs. Improved sensitivities and specificities, close to 100%, were obtained for both sera of patients and dogs. Moreover, high sensitivity and specificity were observed even for canine sera presenting low IFAT anti-*Leishmania* antibody titers or from asymptomatic animals.

**Conclusions/Significance:**

The use of combinations of B cell predicted synthetic peptides derived from antigens A2, NH, LACK and K39 may provide an alternative for improved sensitivities and specificities for immunodiagnostic assays of VL.

## Author Summary

Visceral leishmaniasis is endemic in many areas of tropical and subtropical America where it constitutes a significant public health problem. It is usually diagnosed by enzyme-linked immunosorbent assays (ELISA) using crude *Leishmania* antigens, but a variety of other immunological methods may also be applied. Although these approaches are useful, historically their sensitivity and specificity have often been compromised by the use of complex mixtures of antigens. In this context, the use of combinations of purified, well-characterized antigens appears preferable and may yield better results. In the present study, combinations of peptides derived from the previously described *Leishmania* diagnostic antigens A2, NH, LACK and K39 were used in ELISA against sera from 106 dogs and 44 human patients. Improved sensitivities and specificities, close to 100%, for both sera of patients and dogs was observed for ELISA using some combinations of the peptides, including the detection of VL in dogs with low anti-*Leishmania* antibody titers and asymptomatic infection. So, the use of combinations of B cell predicted synthetic peptides derived from antigens A2, NH, LACK and K39 may provide an alternative for improved sensitivities and specificities for immunodiagnostic assays of VL.

## Introduction

Zoonotic visceral leishmaniasis (VL) caused by *Leishmania infantum* is an important emerging parasitic disease found in countries around the Mediterranean basin, in the Middle East, and in Latin America [1], [2]. In these areas, wild canids constitute major sylvatic reservoirs, and domestic dogs are the principal urban reservoir hosts [3], [4]. Hence, euthanasia of seropositive dogs has been adopted as a mainstay control measure in some countries [5]. However, domestic reservoir control programs may fail because of the high incidence of canine infection, the insensitivity of the diagnostic tests to detect infectious dogs and time delays between diagnosis and **euthanasia** by public health services [4]. Although adopted in European countries, treatment of infected dogs is not allowed in Brazil, based on the assumption that treated dogs may also remain as a source of parasites for sand fly infection. In this context, sensitive diagnostic tests, applicable to field conditions, are becoming increasingly necessary to facilitate and improve the control of disease [6].

Enzyme-linked immunosorbent assays (ELISAs) [7] and indirect fluorescence antibody tests (IFAT) [8] are widely used for serological diagnosis of VL. However, these tests present relative low sensitivity and specificity, which underestimates the actual rate of infection and allows the maintenance of infected animals and transmission. Several defined *Leishmania* antigens have been tested to overcome these difficulties and to improve both sensitivity and specificity [9]. Immunochromatographic tests for the diagnosis of leishmaniasis using the rK39 antigen has been evaluated in several countries, with variable results [6], [10], [11]. Development of effective diagnosis is also critical for control and possible eradication of visceral leishmaniasis and sensitive and specific rapid tests may be especially helpful to achieve this goal [12]. Therefore, there are still much room for improvement of serological diagnosis of VL, including identification and combination antigens and test formats.

B cell epitopes prediction by bioinformatics analysis of protein sequences has been proposed as a good alternative to select peptides for diagnostic tests [13], [14]. In the present study, we tested, in ELISA against sera from 44 patients and 106 dogs, combinations of predicted B cell peptides derived from A2, NH, LACK and K39, which have been previously evaluated as antigens for serodiagnosis of visceral leishmaniasis [15]–[21]. Improved sensitivity for detection of asymptomatic and symptomatic canine visceral leishmaniasis (CVL), including canine sera with low anti-*Leishmania* antibody titers as detected by IFAT, and active disease in human patients was demonstrated for the majority of the peptide combinations.

## Methods

### Ethics Statement

Sera of dogs were obtained from already-existing collections (Sera collection of the Laboratory of Molecular Biology of the Faculty of Pharmacy, Federal University of Minas Gerais). Approval to use the samples was obtained from institutional review board (IRB) - Comitê de Ética em Experimentação Animal (CETEA) from Universidade Federal de Minas Gerais (UFMG), under the protocol 20/2010.

Sera of human patients were also obtained from an already-existing collection (Sera collection of the Laboratory of Immunoparasitology of the Research Center René Rachou, Fundação Oswaldo Cruz). IRB approval to use the samples was obtained from the Institutional Committee on Ethics of Human Research of Fundação Oswaldo Cruz, under the protocol 12/2006. All samples were analyzed anonymously.

### Mapping B-cell Epitopes

The aminoacid sequence of A2 (amastigote stage-specific S antigen homolog of *L. donovani*), k39 (kinesin related protein of *L. chagasi*), LACK (*Leishmania* analogue of the receptor kinase C) and NH (nucleoside hydrolase) proteins were subjected to analysis with software available online at http://www.expasy.org/tools/protscale.html. The analyses generate numerical and graphical scores to predict the position of linear B-cell epitopes. Peptides that fulfilled, at least in part, the criteria of high hydrophilicity (Hoop &Woods), high alpha-helix structures (Chou & Fasman), low coil (Deleage & Roux), low beta-sheet structures (Chou & Fasman), high percentage of accessible residues and low beta-turn structures (Chou & Fasman) were selected for synthesis and screening. The selected peptides were also submitted to BepiPred software (http://www.cbs.dtu.dk/services/BepiPred/) to predict the location of linear B-cell epitopes using a combination of a hidden Markov model and a propensity scale method [13]. BepiPred software scores peptides according to hydrophilicity values, secondary structures and the probability of a aminoacid is located in certain positions as compared to other mapped B cell epitopes. Peptides displaying scores higher than 0.35 may be, therefore, considered putative B cell epitopes.

### Synthetic peptides

Peptides were synthesized according to a standard N-9-ethyloxycarbonyl (Fmoc) strategy on a PSSM8 multispecific peptide synthesizer (Shimadzu, Kyoto, Japan) by solid-phase synthesis and were purified by high performance liquid chromatography and confirmed with a Micromass Q-Tof Micro (Micromass MS Technologies, Division of Waters, Milford, MA) and the peptides obtained by this method were all C-terminal amides.

### Dog sera and infection status

A panel of 106 canine sera was used in the study. Serum samples were divided into three groups based on history of exposure and infection status. Group 1 contained negative control sera from 14 healthy blood donor pets of various ages and breeds (previously classified as seronegative dogs after ELISA-based assays for the detection of antibodies against parasite-specific recombinant antigens rK39, rK26, and rA2) that attended a veterinary clinic in Minas Gerais, Brazil. Group 2 contained 30 serum samples from clinically symptomatic (*n* = 17) and asymptomatic (*n* = 13) dogs in which *L. infantum* visceral infection was proven by the demonstration of the presence of the parasites in bone marrow specimens and/or necropsy tissue samples as previously reported [21]. All infected dogs enrolled in this group were selected during a longitudinal epidemiological survey of CVL carried out in a rural area of endemicity (Pancas, ES; 2003–2004) in southeast Brazil [22]. Group 3 contained sera from 62 dogs with *L. infantum* infection from CVL endemic areas in Brazil. They had been previously tested in IFAT and ELISA. Sera presenting IFAT titers >1∶40 dilutions and ELISA optical densities>cut off values (cut off values were determined by the mean of OD of 14 negative canine control sera plus two standard deviations) were considered positive (IgG) for CVL. All 62 samples had their status confirmed by parasitological analyses which included the search for parasites in bone marrow aspirates by PCR, microscopic examination of Giemsa stained smears and culturing in NNN/LIT medium at 23°C, as previously described [23]. Since a significant correlation was observed between IFAT and ELISA tests for all sera samples (data not shown), positive sera in group 3 were further grouped, according to their previous reactivity in IFAT, regardless its clinical status, as low (*n* = 20) (<1∶320 dilutions), intermediate (*n* = 20) (>1∶320 <1∶640) and high (*n* = 22) (>1∶640) IFAT titers.

### Human sera

Human VL sera were obtained from patients with active visceral leishmaniasis (*n* = 28). Diagnosis of VL was defined when, besides clinical and epidemiologic features, amastigotes were seen at Giemsa stained smears of bone marrow aspirates or promastigote forms were identified on culture of peripheral blood or bone marrow aspirates. In the presence of suggestive clinical and epidemiologic characteristics, negative parasitological findings, but positive anti-*Leishmania* antibodies by IFAT or ELISA, definitive diagnostic was firmed after successful specific treatment. Control sera (*n* = 16) were obtained from individuals living in Vale do Jequitinhonha (in cities: São Pedro do Jequitinhonha, Caju, Virgem das Graças e Melquíades), a rural region of Minas Gerais State in southeast Brazil. None of the individuals presented signs of visceral leishmaniasis at clinical examination. All of them had negative results for specific *Leishmania* PCR in sera samples. Sera samples were also submitted to ELISA with the crude extract of the parasite to confirm that they were negative.

### Enzyme-linked immunosorbent assays ELISA for canine sera

Levels of total IgG immunoglobulin were measured by ELISA. Briefly, 96-well flexible PVC plates (BD Biosciences, San Jose, CA) were sensitized with 5 µg/mL of each synthetic peptide diluted in water (100 µL per well). The sensitized plate was left in the oven until dry and then was left overnight at 4°C. Plates were blocked with PBS-2% casein at 37°C for 1 h and treated successively with 1∶200 dilutions of canine serum samples for 1 h at 37°C. Peroxidase labeled antibodies specific to canine IgG (Sigma, St. Louis, MO) were diluted at 1∶5000 and added for 1 h at 37°C. Following another washing step, the enzyme bound to the immunosorbent is assayed by the addition of a chromogenic substrate, 3,3′,5,5′-tetramethylbenzidine (TMB) in citrate buffer containing hydrogen peroxide. Reactions were stopped by the addition of H_2_SO_4_ 2N. Optical densities were determined at 450 nm in ELISA reader (BioRad, Hercules, CA). Each sera sample was assayed in triplicate. The lower limit of positivity (cut off) was determined by the mean of OD of 14 negative canine control sera plus two standard deviations.

### Enzyme-linked immunosorbent assays ELISA for human sera

Sensitization of the plates followed the same as described above. However, plates were sensitized with 40 µg/mL of each synthetic peptide diluted in water (100 µL per well, resulting in 4 µg/well). When 2 peptides were tested simultaneously (peptides 13 and 47, 13 and 19, 18 and 19, 17 and 47 and 19 and 47), plates were sensitized with 20 µg/mL of each synthetic peptide. After antigen sensitization, the plates were blocked with 2% BSA at 37°C for 2 h and treated successively with 1∶100 dilutions of patients serum samples for 1 h at 37°C. After washing step, biotinilated labeled antibodies human-IgG (Sigma, St. Louis, MO) were diluted at 1∶5000 and added to the plate for 1 h at 37°C. Then, add the streptavidin-peroxidase conjugate diluted 1∶1000 for 30 minutes at 37°C. After three washes, the substrate 3,3′,5,5′-tetramethylbenzidine (TMB) in citrate buffer containing hydrogen peroxide were added to the plate. Reactions were stopped by the addition of H_2_SO_4_ 2N. Optical densities were read at 450 nm in ELISA reader (BioRad, Hercules, CA). Each sera sample was assayed in triplicate. The lower limit of positivity (cut off) was determined by the mean of OD of 16 negative human control sera plus two standard deviations.

### Statistical analysis

One-Way ANOVA test was used to compare the performances of the assays. A *p* value of less than 0.05 was considered significant. Sensitivity and specificity were calculated by binary classification test. The sensitivity and specificity for each test were calculated by using the formulas: Sensitivity = True positive/(True positive+False negative)×100% and Specificity = True negative/(True negative+False positive)×100%.

## Results

### Bioinformatics analysis

The study of the structure of proteins (ProtScale) allowed the selection of five peptides, as shown in Table 1: TPAVQKRVKEVGTKP and TTVVGNQTLEKVT, corresponding to numbers 17 and 18 peptides, respectively, derived from Nucleoside Hydrolase antigen, VVSTSRDGTAISWK, corresponding to peptide 19, derived from LACK protein and ESTTAAKMSAEQDRESTRATLE, corresponding to peptide number 13, derived from K39 protein. Additionally, in Table 1 is represented the peptide derived from the A2 protein, corresponding to peptide 47 (VGPQSVGPLSVGPQSVGPLS). However, the inclusion of this peptide was based on previous analysis of epitope prediction and reactivity with sera of BALB/c mice vaccinated with the A2 antigen [24]. Among the peptides select, 4 (peptides 13, 17, 19 and 47) also showed linear B-cell epitopes with significant values in the analysis by BepiPred, with emphasis on peptides 13 and 47, derived from K39 and A2 antigens, which presented higher scores. By contrast, the peptide 18 showed a low score for the presence of linear B-cell epitopes.

**Table 1:** Bioinformatic analysis of A2, K39, LACK and NH proteins to predict linear B-cell epitopes.

ExPASy	Parameters	Peptide 13	Ppetide 17	Peptide 18	Peptide 19	Peptide 47
Av (Max;Min)/Prot	Alpha-helix	1.1 (0.6; 1.3)	1 (0.7; 1.2)	0.96 (0.7; 1.2)	0.88 (0.7; 1.2)	0.77 (0.7; 1.2)
	Beta-turn	0.9 (0.6; 1.3)	0.83 (0.6; 1.3)	0.9 (0.6; 1.3)	0.91 (0.7; 1.3)	1.13 (0.6; 1.2)
	Beta-sheet	0.85 (0.6; 1.2)	1.1 (0.8; 1.3)	1.1 (0.8; 1.3)	1 (0.7; −1.3)	1 (0.7; 1.4)
	Coil	0.95 (0.8; 1.1)	1 (0.8; 1.2)	0.93 (0.8; 1.2)	0.92 (0.8; −1.1)	1.13 (0.8; 1.15)
	% Ac. residues	6.6 (4.2; 8)	6.4 (4; 7)	6.2 (4; 7)	5.8 (3.6; 7.4)	6.08 (4.2; 7)
	Hydrophilicity	0.73 (−1.2; 2)	0.6 (−1.2; 1.3)	−0.1 (−1.2; 1.3)	0.12 (−1; 1.2)	−0.4 (−1.4; 0.6)

BepiPred	Parameters	Peptide 13	Ppetide 17	Peptide 18	Peptide 19	Peptide 47
Max; Min/aa	Score>0.35	0.12; 1.53	0.33; 1.33	0.03; 0.51	0.01; 1.08	0.69; 1.64

Av: Average, Max: Maximum, Min: Minimum, Prot: Protein, aa: Aminoacid, Ac: Acid.

### Reactivity of dogs and human visceral leishmaniasis sera with the selected peptides

Initially, to test the reactivity of the selected peptides with antibodies present in dogs' sera with VL, the spot synthesis technique, followed by immunoassay was applied (data not shown). The membranes were incubated with a pool of sera from infected and control animals. Four of the five peptides showed high intensity reactions with sera of dogs with confirmed VL as compared with the control group. Peptide 19 presented reactivity with both positive and negative sera. However, considering the use of pooled sera on the spot synthesis experiment, peptide 19 was also included in a more discriminatory analysis through ELISA with individual sera samples.

**Figure 1:**
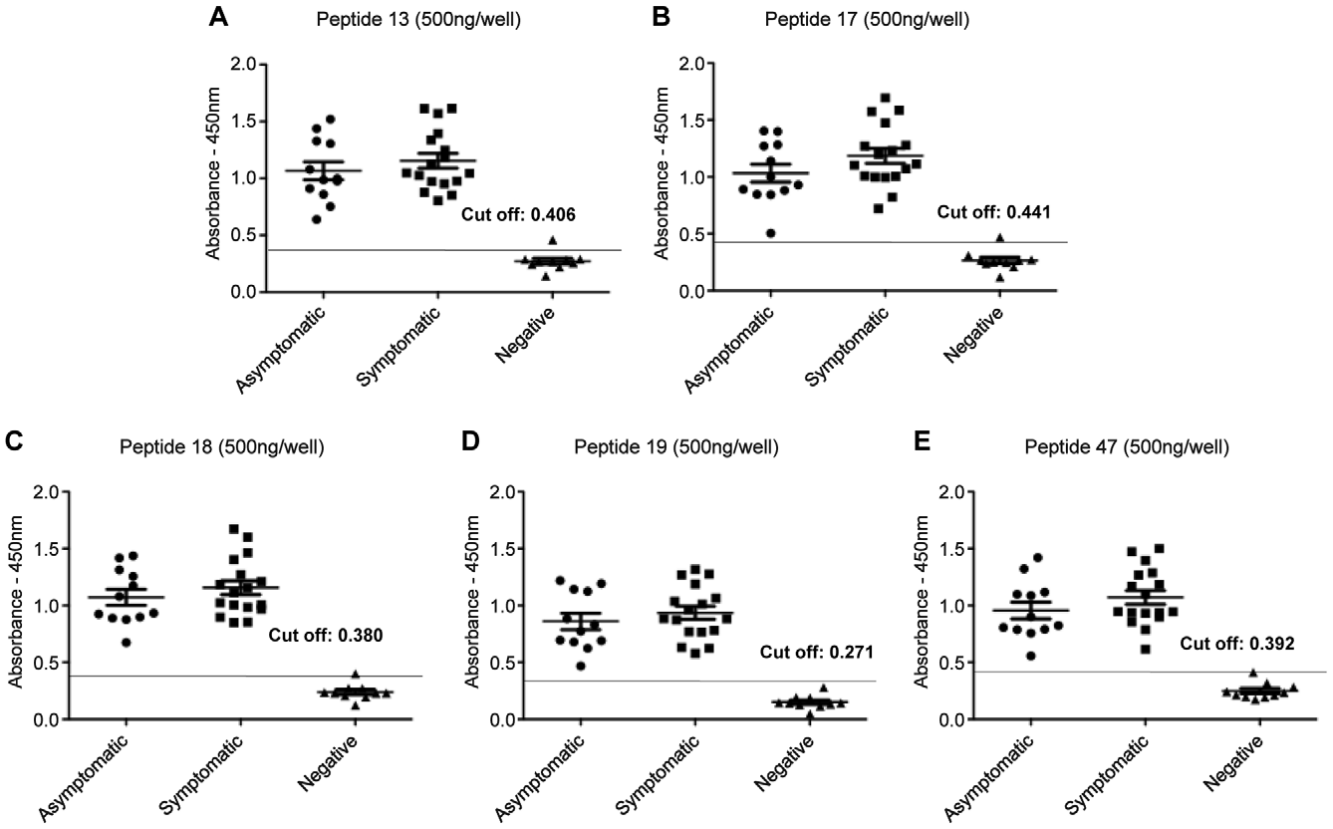
ELISA with individual synthetic peptides for detection of IgG-antibodies in dogs with *L. infantum* infection. Detection of anti-leishmanial total IgG antibodies with synthetic peptides by ELISA assay using sera from asymptomatic (*n* = 13) and symptomatic (*n* = 17) dogs and control group (*n* = 14). It was used 500 nanograms/well of peptides 13 (panel **A**), 17 (panel **B**), 18 (panel **C**), 19 (panel **D**) and 47 (panel **E**). The sensitivity and specificity of asymptomatic and symptomatic dogs ranged between 100 and 90%, respectively, for both.

All peptides were then tested against Group 2 sera, which included samples collected from symptomatic and asymptomatic animals (Figure 1). As shown in figure 1, all peptides were able to detect as positives all the sera samples from both asymptomatic (*n* = 13) and symptomatic animals (*n* = 17) Figure 1. No significant differences were observed in sensitivity between the two groups.

**Figure 2:**
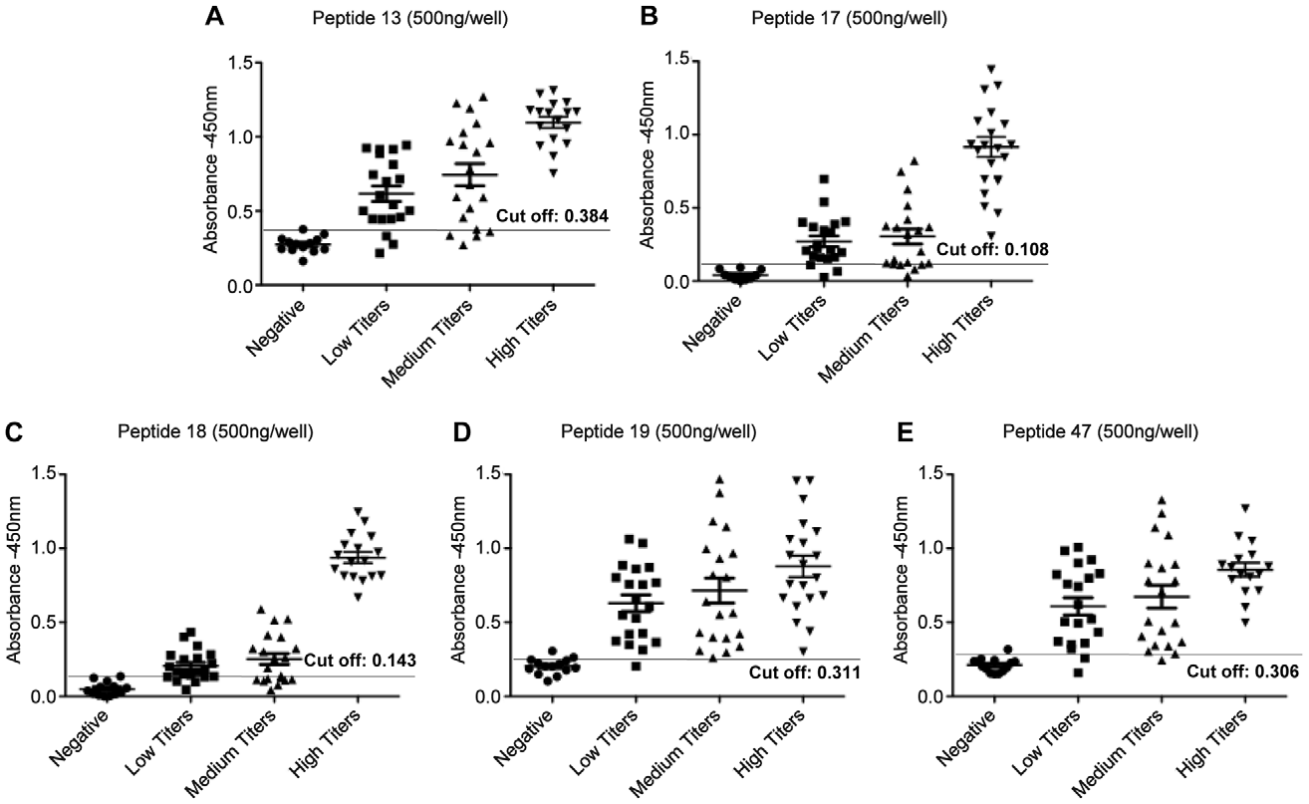
Reactivity of sera from infected dogs displaying different IFAT antibody titers with peptides. Detection of anti-leishmanial total IgG antibodies with synthetic peptides by ELISA assay using canine samples classified according to IFAT reactivity as low (<1∶320 dilutions), intermediate (>1∶320 <1∶640) and high (>1∶640) titers and 500 nanograms/per well of peptides 13 (panel **A**), 17 (panel **B**), 18 (panel **C**), 19 (panel **D**) and 47 (panel **E**).

The reactivity of the 5 peptides was further evaluated with a larger panel of sera from parasitological positive (Group 3) or control dogs (Group 1), which included sera samples classified according to IFAT reactivity as low (<1∶320 dilutions), intermediate (>1∶320 <1∶640) and high (>1∶640) titers (Figure 2). High sensitivity (100%) was observed for all peptides to detect infection in dogs with high IFAT antibody titers (22 animals), when the peptides were tested individually (Figure 2/Table 2). However, decreased sensitivities (varying between 55% and 90%) were observed for all peptides when tested against sera of dogs with IFAT intermediate (>1∶320 <1∶640) (20 animals) or low antibody titers (1∶80 >1∶320) (20 animals) (Table 2). Concerning specificity, a value of 100% was observed for peptide 17 and 92% for the other peptides, as shown in Table 2.

**Table 2:** Performance of ELISA employing synthetic peptides and canine sera classified according to IFAT reactivity.

Peptides	Low Titer* (n = 20)	Intermediate Titer (n = 20)	High Titer (n = 22)	Uninfected Dogs (n = 14)
	Se (%)	Se (%)	Se (%)	Sp (%)
**13**	85	75	100	92
**17**	85	75	100	100
**18**	70	55	100	92
**19**	90	85	100	92
**47**	90	85	100	92
**13 and 47**	95	85	ND	100
**13 and 19**	95	95	ND	100
**18 and 19**	95	90	ND	100
**47 and 17**	90	80	ND	100
**47 and 18**	95	95	ND	100

Se: sensitivity, Sp: specificity.

*: canine sera samples were classified according to IFAT reactivity as low (<1∶320 dilutions) and intermediate (>1∶320 <1∶640) titers and high (>1∶640) antibody titers.

**Figure 3:**
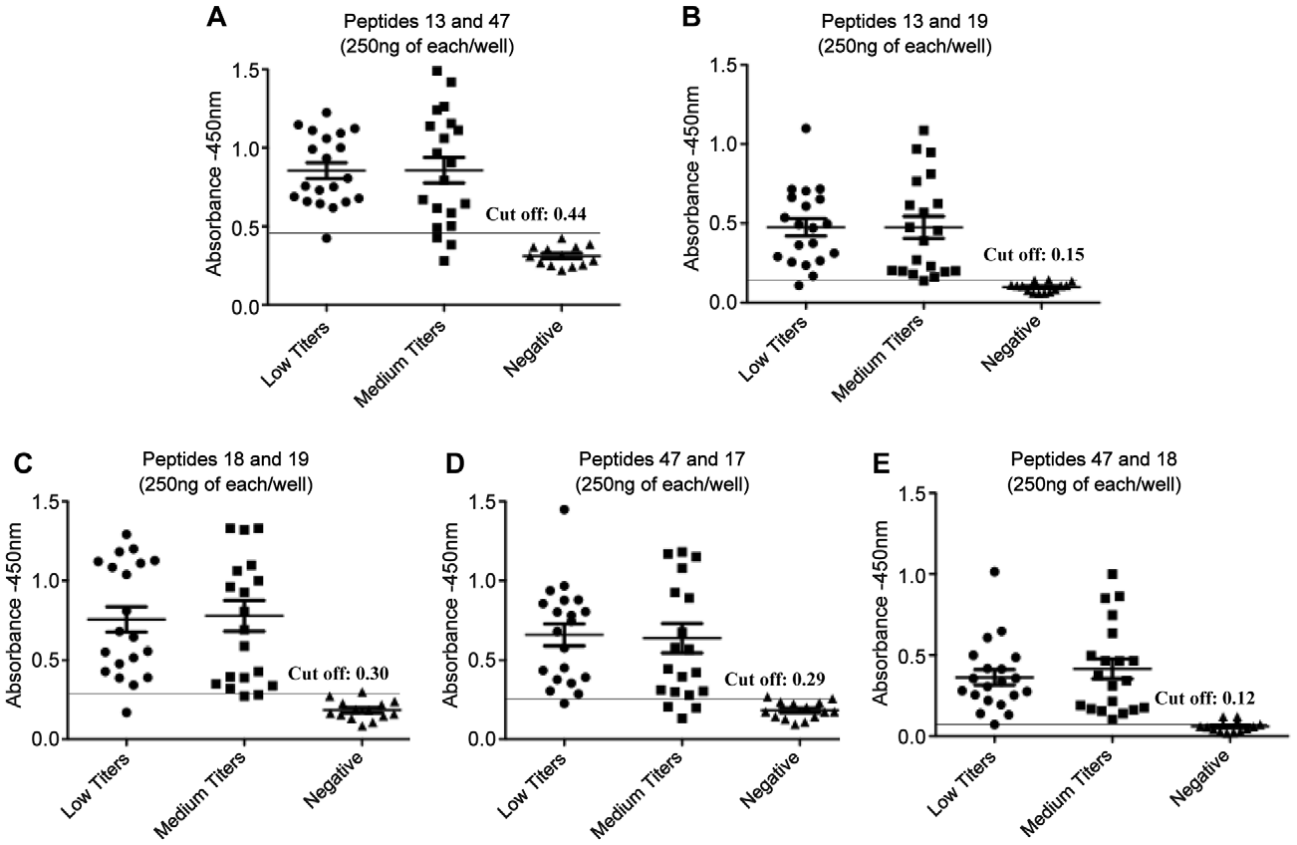
Reactivity of sera from infected dogs displaying different antibody titers with combined peptides. Detection of anti-leishmania total IgG antibodies with synthetic peptides by ELISA assay using canine samples classified according to IFAT reactivity as low (<1∶320 dilutions) and intermediate (>1∶320 <1∶640) titers and 250 nanograms/per well from each peptide. Peptides were combined in pairs in the same reaction as following: peptides 13 and 47 (panel **A**), 13 and 19 (panel **B**), 18 and 19 (panel **C**), 47 and 17 (panel **D**) and 47 and 18 (panel **E**).

Since decreased sensitivity was detected for each peptide individually with sera of dogs with intermediate and low IFAT titers, we tested the hypothesis that the sensitivity to detect VL would increase by combining peptides in the same reaction. Assuming this would increase sensitivity by broaden instead of simply increasing the number of available epitopes for reaction, half of the concentration was used for each peptide instead of double the total peptide concentration. Combinations of two peptides were then tested against dog sera with low and intermediate antibody titers (Figure 3/Table 2). For sera with low antibody titers, the best results, i.e, improved sensitivities as compared to peptides tested individually, ranged from 90% to 95%, were obtained with combinations between the peptides 13 and 47, 13 and 19, 18 and 19, 47 and 17 and 47 and 18. Specificity was also improved, reaching 100% for all of these combinations. The sensitivity of peptide 47 was not altered when combined with peptide 17, which in contrast, had its sensitivity improved from 85% to 90% (Figure 3/Table 2). For sera with intermediate antibody titers, improved sensitivities for both peptides were observed for combinations between peptides 13 and 19, 18 and 19, 47 and 18, varying from 80% to 95%. The sensitivity of peptide 47 (85%) was not affected when combined with peptide 13 and decreased when associated to peptide 17 (Figure 3/Table 2).

**Figure 4:**
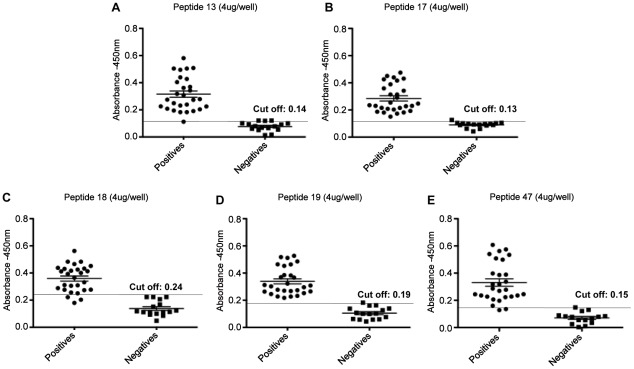
Detection of IgG-antibodies in sera of human patients with visceral leishmaniasis with individual peptides. Detection of anti-leishmania total IgG antibodies with synthetic peptides by ELISA assay using sera from patients with active visceral leishmaniasis (*n* = 28) and healthy individuals with previous negative results in ELISA to *Leishmania* (*n* = 16) and 4 µg/per well peptides 13 (panel **A**), 17 (panel **B**), 18 (panel **C**), 19 (panel **D**) and 47 (panel **E**).

**Figure 5:**
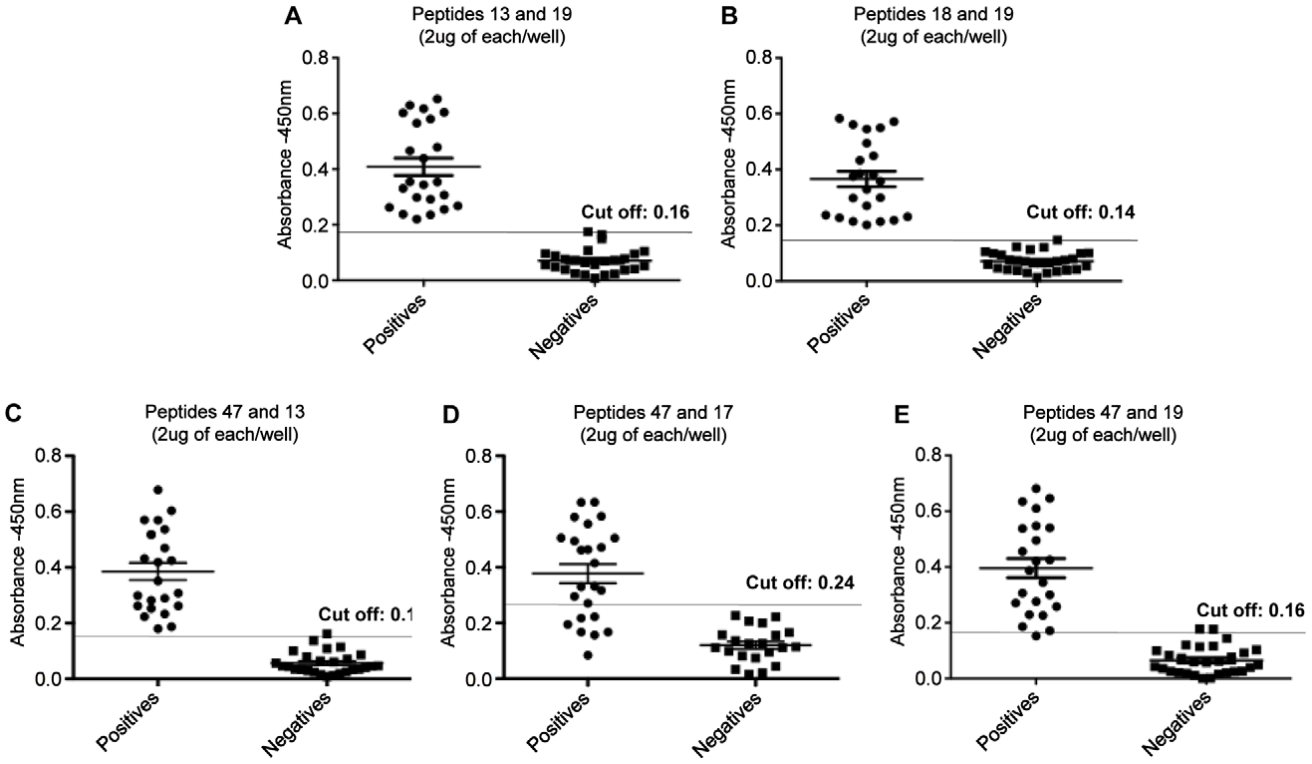
Detection of IgG-antibodies in sera of human patients with visceral leishmaniasis with combined peptides. Detection of anti-leishmania total IgG antibodies with synthetic peptides by ELISA assay using sera from patients with active visceral leishmaniasis (*n* = 28) and healthy individuals with previous negative results in ELISA to *Leishmania* (*n* = 16) and 2 µg/per well from each peptide. Peptides were combined in pairs in the same reaction as following: peptides 13 and 19 (panel **A**), 18 and 19 (panel **B**), 47 and 13 (panel **C**), 47 and 17 (panel **D**) and 47 and 19 (panel **E**).

Peptides were also tested (individually and combined) against human sera (*n* = 44) including patients with active visceral leishmaniasis (*n* = 28) and healthy individuals with previous negative results in ELISA to *Leishmania* (*n* = 16). The results obtained are shown in Figure 4 and 5/Table 3. Sensitivity values of 82%, 93% and 96% were observed for peptides 18, 47 and 13, respectively. And ELISA with peptides 17 and 19 gave the best results, displaying sensitivities of 100%. The specificity for the peptides tested individually ranged from 81% to 94%. The combination of peptides brought an improvement in sensitivity and specificity between peptides 13 and 19, 18 and 19, 47 and 13, and 19 and 47 where we observe a sensitivity of 100% for the first three combinations and 95.65% for the last one, respectively. Only for the combination between peptides 17 and 47 a reduction in sensitivity to 70.83% was observed. Specificity values for all combination ranged from 93.10% to 100% (Figure 4 and 5/Table 3).

**Table 3:** Performance of ELISA employing synthetic peptides and sera of patients with visceral leishmaniasis.

Total IgG	Acute phase of infection (n = 28)	Healthy Control Individuals (n = 16)
	Se (%)	Sp (%)
**Peptide 13**	96	81
**Peptide 17**	100	94
**Peptide 18**	82	81
**Peptide 19**	100	94
**Peptide 47**	93	94
**Peptides 13 and 19**	100	93
**Peptides 18 and 19**	100	100
**Peptides 13 and 47**	100	96
**Peptides 17 and 47**	71	100
**Peptides 19 and 47**	97	93

Se: sensitivity, Sp: specificity.

## Discussion

In the present work, the *Leishmania* proteins A2, K39, LACK and NH were submitted to B cell epitope prediction and the derived synthetic peptides were evaluated through ELISA against sera of dogs and patients for the serodiagnosis of VL. Using the Protscale software, six different parameters were evaluated for each protein to select peptides. Considering the scores for these parameters, an adequate profile was observed for the majority of peptides, as compared to the minimal and maximum scores for the corresponding proteins, except for peptide 47. Peptide 47 displayed the lower values for hydrophilicity and presence of alpha helix, which are expected to be high for B cell epitopes, and the highest values for coil and beta turn structures, which in contrast are expected to be low. On the other hand, prediction using BepiPred resulted in scores higher than 0.35 for all peptides, except for peptide 18. Altogether, our results indicate that the two analyses may be complementary to each other and that this strategy is useful for selecting diagnostic antigens.

Accurate diagnosis of canine leishmaniasis is essential towards a more efficient control of this zoonosis, but it remains problematic due to the high incidence of asymptomatic infections [25]. Initially, we tested the five peptides with sera from dogs clinically classified as asymptomatic and symptomatic (Group 2). It is noteworthy that the sera samples of Group 2 have been previously tested in ELISA using SLA or the recombinant proteins rA2, rK39 and rK26 [21]. The retrospective analysis of the data obtained by Porrozzi et al. (2007) revealed that 4, 9, 5 and 4 out of 13 sera from asymptomatic animals included in the present study, were not reactive with SLA, rA2, rK39 and rK26, respectively, whereas all the symptomatic samples were positive when either rK26, rK39 or SLA were used as antigens. Using rA2, 6 symptomatic sera samples were identified as negative. Moreover, the majority of samples that were not reactive with these antigens were obtained from asymptomatic animals presenting low antibody titers in IFAT (≤1∶80). In the present analysis, for both asymptomatic and symptomatic VL canine sera, sensitivities and specificities of 90% and 100%, respectively, were observed. Therefore, improved sensitivity was observed for assays using the synthetic peptides as compared to SLA and the recombinant proteins, especially for sera of asymptomatic animals. In this sense, our results largely confirm and improve the potential of these antigens for serodiagnosis of leishmaniasis.

Detection of infection in animals with low or intermediated anti-*Leishmania* antibody titers, regardless their clinical status, is critical for diagnosis and control of VL. The failure to detect infection in these animals may contribute to the maintenance of parasite's transmission for both canine and human populations, one of the major factors that hinder control strategies. On the other hand, highly sensitive diagnosis may require combined antigens. As indicated by immunoproteomic approaches, *Leishmania* parasites display extensive variability in antigenic composition, and apparently absence of immunodominant antigens when individual sera samples are analyzed [26], suggesting that single antigen diagnostic tests may display decreased sensitivities. Indeed, rK39 based tests may lack sensitivity for canine sera with low antibody concentration [19].

By combining two peptides, increased sensitivities (90–95%) and specificity (100%) were observed for dog sera with low IFAT antibodies titers. Similar findings were also observed for sera with intermediated antibodies titers (80–95% of sensitivity and 100% specificity). Improved sensitivities may have resulted from increased number of reactive epitopes, leading to increased OD readings and numbers of positive sera as compared to the reactivity with a single peptide. These findings will be particularly useful for diagnosis of dogs with low and intermediate titers of antibodies, since most current tests fail in this task.

Since early and sensitive diagnosis is seen as a critical aspect for management and, possibly, eradication of human visceral leishmaniasis [12], [27]–[29], we have also investigated the reactivity of the peptides with sera of patients with active VL. Similarly, improved results have been observed when the combinations of peptides were tested against sera of human patients with active disease, suggesting that the epitopes selected were also recognized individually by human sera and that their serological reactivity may be independent and complementary, leading to an additive effect. Therefore, the association of peptides is an alternative to broaden the epitopes to be detected by antibodies, improving sensitivity. On the other hand, the absence of improved sensitivity for association between the peptides 17 and 47 may be explained by the presence of low levels of *Leishmania* specific antibodies in the control negative sera, since healthy controls were selected from endemic area and previous exposure of these individuals to parasite antigens may not be completely ruled out.

In many endemic areas, VL frequently overlaps with the occurrence of other forms of leishmaniasis or even with other infectious diseases, such as tuberculosis and leprosy. Cross-reactivity with antibodies raised against other infectious diseases consists in an additional shortcoming for development of specific visceral leishmaniasis diagnosis. Cross-reactivity of synthetic peptides with sera of patients presenting other infections was not assessed in the present work. Therefore, additional investigations are further warranted to better determine peptides specificity.

In conclusion, the combination of synthetic peptides, identified through B cell epitope predicition, may be useful for the development of highly sensitive and specific serodiagnosis for VL. The peptides identified may be especially interesting for the development sensitive immunochromatographic tests. Since these test format do not require sophisticated laboratory facilities or trained personnel staff to be routinely performed, and antibody quantification is not required for diagnosis of VL, they are more practical and easily applied, allowing rapid diagnosis in field conditions in endemic areas of difficult access to laboratory facilities [11], [12], [30]–[32]. Therefore, these peptides coupled to immunochromatographic tests may allow sensitive and early detection of infected dogs and their fast withdraw from transmission areas, regardless their antibody levels and clinical status, improving the control of VL in endemic areas.
